# Atypical hemolytic uremic syndrome in the setting of complement-amplifying conditions: case reports and a review of the evidence for treatment with eculizumab

**DOI:** 10.1007/s40620-016-0357-7

**Published:** 2016-11-15

**Authors:** Arif Asif, Ali Nayer, Christian S. Haas

**Affiliations:** 1 Department of Medicine, Jersey Shore University Medical Center, Hackensack-Meridian Health, Seton Hall-Hackensack-Meridian School of Medicine, 1945 NJ Route 33, Neptune, NJ 07753 USA; 2 Division of Nephrology and Hypertension, Miller School of Medicine, University of Miami, Batchelor Research Institute (R762), 1580 N.W. 10th Avenue, Miami, FL 33136 USA; 3 Division of Nephrology, Dialysis and Transplantation, Department of Medicine I, University of Lübeck, Ratzeburger Allee 160, 23562 Lübeck, Germany

**Keywords:** Complement, Thrombotic microangiopathy, Pregnancy, Hypertension, Kidney transplantation

## Abstract

Atypical hemolytic uremic syndrome (aHUS) is a rare, genetic, progressive, life-threatening form of thrombotic microangiopathy (TMA) predominantly caused by dysregulation of the alternative pathway of the complement system. Complement-amplifying conditions (CACs), including pregnancy complications [preeclampsia, HELLP (hemolysis, elevated liver enzymes, low platelet count) syndrome], malignant hypertension, autoimmune diseases, transplantation, and others, are associated with the onset of TMA in up to 69 % of cases of aHUS. CACs activate the alternative pathway of complement and may be comorbid with aHUS or may unmask a previously undiagnosed case. In this review, three case reports are presented illustrating the onset and diagnosis of aHUS in the setting of different CACs (pregnancy complications, malignant hypertension, renal transplantation). The report also reviews the evidence for a variety of CACs, including those mentioned above as well as infections and drug-induced TMA, and the overlap with aHUS. Finally, we introduce an algorithm for diagnosis and treatment of aHUS in the setting of CACs. If TMA persists despite initial management for the specific CAC, aHUS should be considered. The terminal complement inhibitor eculizumab should be initiated for all patients with confirmed diagnosis of aHUS, with or without a comorbid CAC.

## Introduction

Thrombotic microangiopathy (TMA) is a life-threatening syndrome of systemic microvascular occlusions and is characterized by sudden or gradual onset of thrombocytopenia, microangiopathic hemolytic anemia, and renal or other end-organ damage [1, 2]. TMA has been associated with diverse diseases and syndromes, such as systemic infections, cancer, pregnancy complications [e.g. preeclampsia, eclampsia, HELLP (hemolysis, elevated liver enzymes, low platelet count) syndrome], autoimmune disorders [e.g. systemic lupus erythematosus (SLE), systemic sclerosis, antiphospholipid syndrome], hematopoietic stem-cell or organ transplantation, and severe hypertension [1].

The etiologies of TMA also include atypical hemolytic uremic syndrome (aHUS) [1], a rare, progressive, life-threatening form predominantly caused by dysregulation of the complement alternative pathway [3]. aHUS can manifest at any age. While approximately 80 % of patients present with thrombocytopenia, microangiopathic hemolytic anemia, and renal impairment [4], onset may be more gradual in other patients [5]. Because aHUS can affect multiple vascular beds [6], extrarenal manifestations occur in up to 48 % of patients, with frequent neurologic and cardiovascular involvement [7–10].

Patients with aHUS who are untreated remain at lifelong risk of renal impairment, end-stage renal disease, extrarenal complications, and premature death [4, 9]. Management with plasma exchange/plasma infusion (PE/PI) may improve hematologic parameters temporarily [11, 12] but not long-term outcomes [4]. The efficacy and safety of eculizumab (Soliris®, Alexion Pharmaceuticals, Inc., Cheshire, CT, USA), a terminal complement inhibitor and the only approved treatment for aHUS [13, 14], were first established in two prospective, multicenter clinical studies [15, 16], followed by prospective, multicenter studies in pediatric [17] and adult [18] populations. Eculizumab therapy was demonstrated to inhibit complement-mediated TMA and improve hematologic parameters, renal function, and quality of life [15, 17, 18].

**Fig. 1:**
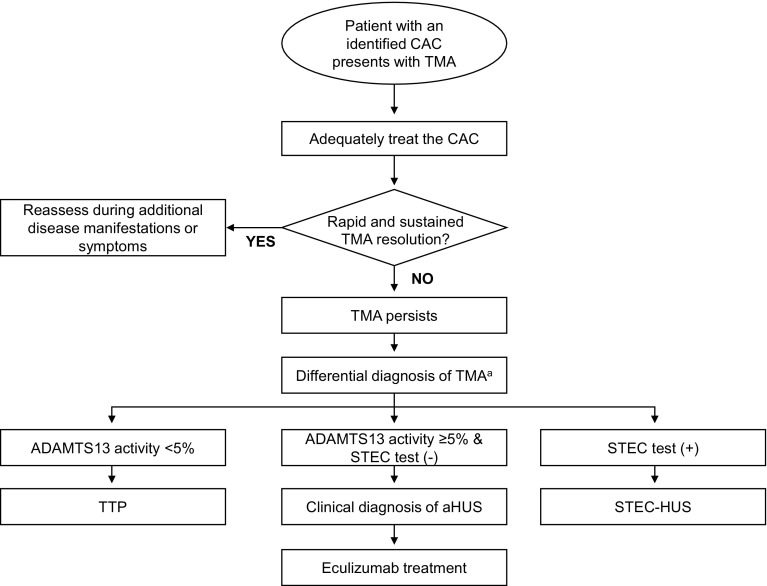
Management algorithm for patients with CACs and TMA. *ADAMTS13* a disintegrin and metalloproteinase with a thrombospondin type 1 motif, member 13, *aHUS* atypical hemolytic uremic syndrome, *CAC* complement-amplifying condition, *STEC* Shiga-like toxin-producing *Escherichia coli, TMA* thrombotic microangiopathy, *TTP* thrombotic thrombocytopenic purpura. ^a^The differential diagnosis section of the algorithm has been adapted from [5]

According to the “multiple-hit” hypothesis [19], aHUS is a consequence of both genetic predisposition to alternative complement dysregulation as well as the occurrence of events or conditions that may precipitate TMA by activating complement and/or damaging the endothelium [19, 20]. Complement-amplifying conditions (CACs), such as pregnancy complications (preeclampsia, HELLP), autoimmune diseases and others, may be comorbid with aHUS, unmask a previously undiagnosed case, or lead to a misdiagnosis [3, 21–23]. Malignant hypertension (MHT) is another CAC that may precipitate aHUS or occur secondary to aHUS [21], potentially confounding the diagnosis. In this review, we describe case reports that demonstrate the onset of aHUS in the setting of CACs. We also review the evidence for a number of CACs, including pregnancy complications, MHT, autoimmune diseases, transplantation, infections, and drugs, and the overlap of these disorders with aHUS. Finally, we present an algorithm for diagnosis and treatment of aHUS in the setting of CACs (Fig. 1) [5].

## Case reports

### Case 1

A 33-year-old Hispanic woman developed abruptio placentae leading to fetal death at 33 weeks of gestation. She underwent cesarean section and hysterectomy, and a subsequent exploratory laparotomy. The patient had extensive blood loss and received numerous transfusions. She developed thrombocytopenia [39 × 10^9^/L (normal range 150–350 × 10^9^/L)], microangiopathic hemolytic anemia [hemoglobin level 6.7 g/dL (normal range 14.0–17.5 g/dL)]; lactate dehydrogenase (LDH) level, 2670 U/L (normal range at institution, 100–200 U/L); haptoglobin level, 5.8 mg/dL (normal range at institution, 26–185 mg/dL); numerous schistocytes on a blood smear, and renal failure [serum creatinine level, 6.0 mg/dL (normal range 0.6–1.2 mg/dL)] necessitating initiation of hemodialysis. The fibrinogen level as well as prothrombin and partial thromboplastin times were normal. ADAMTS13 (a disintegrin and metalloproteinase with a thrombospondin type 1 motif, member 13) activity testing was ordered and PE was initiated. The patient showed minimal improvement in hematologic parameters (hemoglobin level, 7.0 g/dL; platelet count, 42 × 10^9^/L) and no improvement in renal function (dialysis dependent) after five daily PEs, and the ADAMTS13 activity level was 56 %. Following diagnosis of aHUS, PE was discontinued. After the discontinuation of PE, the patient was vaccinated against meningococcus, antibiotic prophylaxis was started, and eculizumab therapy was initiated. Two weeks later, dialysis was discontinued. Laboratory tests showed a platelet count of 147 × 10^9^/L, hemoglobin level of 8.8 mg/dL, and serum creatinine level of 3.4 mg/dL. At last follow-up after 27 weeks of eculizumab therapy, platelet count (198 × 10^9^/L), hemoglobin level (13.0 g/dL), and serum creatinine level (0.9 mg/dL) were normal. The patient remains on ongoing eculizumab therapy.

### Case 2

A 43-year-old Caucasian woman with a history of migraine headaches since childhood presented with severe headaches and visual impairment lasting for several days. The examination showed a blood pressure of 300/185 mmHg resulting in immediate hospitalization. Fundoscopic examination revealed papilledema, and a subsequent cerebral magnetic resonance tomography showed alterations consistent with posterior reversible encephalopathy syndrome. Laboratory tests including hemoglobin level of 10.8 g/dL, LDH level of 447 U/L (normal range at institution, <250 U/L) and schistocytes on a blood smear revealed microangiopathic hemolytic anemia; the platelet count was normal. Acute kidney injury [serum creatinine level, 3.4 mg/dL (normal range at institution, 0.5–1.0 mg/dL); proteinuria] also was evident. PE was initiated because thrombotic thrombocytopenic purpura (TTP) could not be ruled out initially, but was discontinued after the ADAMTS13 activity was determined to be 64 %. The patient’s hypertension was managed with intravenous and oral antihypertensive medications resulting in the resolution of neurological symptoms. Stool examination showed no Shiga toxin-producing *Escherichia coli* (STEC). A kidney biopsy revealed severe obliterative arteriolosclerosis, ischemic glomerular collapses, and extensive acute tubular injury. Together with typical signs of hypertensive retinopathy and echocardiographic evidence of hypertensive heart disease, the patient was considered to have MHT. However, despite adequate blood pressure control and resolution of hemolysis (LDH, 163 U/L), there was no improvement in anemia (hemoglobin, 10.7 g/dL) and renal function (serum creatinine level, 3.3 mg/dL) over approximately 5.5 weeks from presentation. Therefore, aHUS was diagnosed with MHT as a presenting sign. No complement gene mutations were identified. After meningococcal vaccination and antibiotic prophylaxis, initiation of eculizumab therapy resulted in gradual improvement of renal function. After 9 months of therapy, the patient’s hemoglobin level was 12.2 g/dL and serum creatinine level was stable at 2.1 mg/dL. After 11 months, the hemoglobin and serum creatinine levels were 12.9 g/dL and 2.0 mg/dL, respectively. The patient discontinued from eculizumab therapy after 1 year.

### Case 3

A 37-year-old Caucasian female hemodialysis patient with a 14-month history of end-stage renal disease due to recurrent pyelonephritis underwent living-related donor kidney transplantation. Excellent graft function was noted immediately following the surgery, and the serum creatinine level decreased to 0.9 mg/dL. Over the subsequent days, however, urine output gradually decreased and serum creatinine levels increased (1.85 mg/dL on day 5 post-surgery). Humoral rejection was suspected (increasing titer of donor-specific antibodies), and the patient was treated with high-dose corticosteroids and PI. However, the patient developed anuria. Doppler ultrasound showed near-absent graft perfusion. In addition, TMA was suggested by laboratory values including the presence of schistocytes, platelet count of 33 × 10^9^/L, hemoglobin level of 11.7 g/dL, LDH of 675 U/L (normal range at institution, <250 U/L), serum creatinine of 3.5 mg/dL, and heavy proteinuria (6701 mg/g creatinine). The patient was started on hemodialysis because of volume overload and progressive renal dysfunction. On post-transplant day 8, a diagnosis of aHUS was made. Eculizumab therapy, along with antibiotic prophylaxis for meningococcal infection, was initiated, leading to gradual resolution of hemolysis and improved renal function. A renal allograft biopsy revealed TMA consistent with the clinical diagnosis of aHUS. Immunostaining demonstrated C4d staining of peritubular capillaries consistent with humoral rejection. Immunoabsorption was performed for 3 days followed by two doses of intravenous immunoglobulins. Eculizumab treatment was continued with improvement in renal function without the need for further renal replacement therapy. The patient received meningococcal vaccination following discharge. At a follow-up of 6 months, platelet count continues to be stable at 213 × 10^9^/L, hemoglobin level at 11.9 g/dL, LDH level at 273 U/L, and serum creatinine level at 1.7 mg/dL. The patient continues to receive eculizumab therapy. Genetic testing did not reveal any complement gene mutations.

## Discussion

These case reports illustrate aHUS in the setting of three CACs: pregnancy complications, MHT, and renal transplantation. In all three cases, a CAC preceded the onset of TMA. Importantly, the standard management of the individual CAC (i.e. cesarean section and subsequent hysterectomy after pregnancy complications, antihypertensive medications for MHT, and corticosteroid therapy for humoral allograft rejection) did not resolve TMA. Each patient had a thorough evaluation for potential underlying causes of TMA. After prompt diagnosis of TMA and recognition of aHUS in each case, treatment with eculizumab was associated with improvement in both hematologic parameters and renal function.

Accumulating evidence shows that patients with underlying complement dysregulations are particularly prone to develop TMA when experiencing a CAC. Chronic complement dysregulation, both in aHUS and other disorders, leaves patients predisposed to TMA [24]. When patients are unable to regulate complement, onset or exacerbation of CACs may precipitate aHUS or cause additional manifestations, resulting in persistent TMA despite treatment of CAC symptoms [25]. Findings from a large observational study of patients with aHUS showed that 69 % of the patients had their first TMA manifestations while experiencing a CAC [9].

Proper diagnosis may be particularly challenging in the setting of aHUS and CACs due to overlapping comorbidities [1]. Patients may not necessarily present with the classic triad of microangiopathic hemolytic anemia, thrombocytopenia, and renal impairment [3]; in particular, thrombocytopenia may be absent or mild in MHT [26]. In a large observational study of patients with aHUS, 16 % of patients did not have thrombocytopenia at disease onset [4]. In the described case with MHT, the patient had a normal platelet count at presentation. It is possible that some patients may develop thrombocytopenia relative to earlier laboratory tests, although all values may remain in the normal range. Elevated LDH levels and the presence of schistocytes may also be considered important diagnostic features of microangiopathic hemolytic anemia [5].

### Review of CACs

#### Pregnancy complications

TMA occurs in approximately 1 per 25,000 pregnancies [27]. Pregnancy-related aHUS (P-aHUS) may account for approximately 7 % of total aHUS cases [9] and up to 20 % of cases in adult females [4, 28]. Complement activation may be augmented during pregnancy, when the placenta may be subject to attack by the complement and immune system [28]. In addition, the complement pathway may be activated postpartum due to maternal circulation of fetal cells, infections, and hemorrhage [28]. Recently, increased complement activation was identified in a subset of women with preeclampsia and HELLP syndrome [29].

In addition to microangiopathic hemolytic anemia, thrombocytopenia, and renal insufficiency, general signs and symptoms of P-aHUS may include fatigue, headache, nausea, and vomiting. Diagnosis may be difficult because of similarities between P-aHUS and more common pregnancy complications such as preeclampsia and HELLP [27, 30]. A recent study of 21 women with P-aHUS showed that most cases occurred postpartum and during second pregnancies [28]. Clinical conditions could rapidly deteriorate, resulting in poor maternal outcomes [27]. Hypertension and chronic kidney disease were frequent long-term complications [27]. End-stage renal disease occurred in 76 % of patients. In severe cases, death may occur within hours to days after the onset of P-aHUS [31].

P-aHUS case reports were first published more than 40 years ago [32]. Delmas et al. [33] were the first to show the beneficial effects of eculizumab on hematologic and renal parameters in a patient with postpartum aHUS. More recent case studies also documented the efficacy of eculizumab in the treatment of P-aHUS, including normalization of hematologic parameters and renal function (Table 1) [33–41].

**Table 1:** Cases of aHUS and comorbid CACs treated with eculizumab

Publication	Case description and treatment	Outcomes
Pregnancy complications		
Ardissino et al. [34]	26-year-old female, diagnosed 2 years prior with aHUS, presented at week 17 of gestation with severe hypertension; laboratory values indicated active TMA (low platelets, elevated LDH, 6 % schistocytes) She received 29 PEs over 6 weeks and condition improved, but at 26 weeks of gestation, her platelet count declined despite additional PE; hematologic investigations indicated complement dysregulation Genetic testing results indicated a homozygous *CFH* mutation She received 1 dose of 900 mg IV eculizumab, a second dose 1 week later, and continuous dosing every 2 weeks until delivery	Her condition and laboratory values began to normalize 1 day after the first dose of eculizumab Her pregnancy proceeded uneventfully and she delivered a healthy newborn
Carr et al. [35]	20-year-old female, 7 days post-cesarean delivery, presented with bilateral lower extremity edema, malaise, and bruising Patient had low hemoglobin and platelets, elevated serum creatinine and LDH, 2 + schistocytes, ADAMTS13 100 % Kidney biopsy revealed TMA and acute tubular necrosis PE and prednisone treatment were initiated; after 7 days, she had a partial hematologic response but her renal condition worsened Hemodialysis was initiated and a diagnosis of aHUS made; genetic testing results indicated a mutant allele in the *CFH* gene Eculizumab was initiated (900 mg IV for 4 weeks, then 1200 mg IV continuously every 2 weeks)	Her hematologic condition normalized after 2 weeks and hemodialysis terminated after 6 weeks, renal function normalized after 12 weeks Patient discontinued eculizumab after 9 months Presented 6 months later with similar symptoms at initial presentation She required hemodialysis and eculizumab was restarted Her condition improved and hemodialysis was discontinued 3 weeks after restarting eculizumab
Delmas et al. [33]	26-year-old female admitted 1 week after first delivery with elevated serum creatinine and LDH levels, low platelets and hemoglobin, 9 % schistocytes Family history of aHUS and genetic testing indicated heterozygous mutations in *CFH* and *CFI* genes PE was initiated with some improvement in hematologic but not renal condition; hemodialysis was initiated 3 days after admission, she received 900 mg eculizumab and received a second dose 1 week later; daily PE was reinitiated without supplemental eculizumab 39 days after admission, eculizumab was resumed (1200-mg dose) due to decreasing platelets Eculizumab was administered whenever the CAE assay value was >0.5 U/mL (<0.5 U/mL correlates to total complement blockade)	Eculizumab was tapered from 18 months post-admission She had no signs of aHUS at follow-up 2 months after interrupting eculizumab First reported case of post-partum aHUS treated with eculizumab
Zschiedrich et al. [36]	31-year-old female admitted 3 days after delivery with hypertension, thrombocytopenia, delirium, acute oliguric renal failure; hematology indicated intravascular hemolysis and schistocytes Patient received PE, prednisone, and hemodialysis After 18 days with 27 PE and 9 dialysis sessions, her platelets remained low and serum creatinine elevated Eculizumab was initiated and genetic testing identified a novel mutation in *CFI*	She had full clinical resolution of TMA and favorable renal outcome with eculizumab
Canigral et al. [37]	32-year-old female developed severe bleeding after cesarean delivery that required hysterectomy Laboratory findings included anemia with schistocytes, low platelet count, and elevated serum creatinine, LDH, and urea levels No response to PE and steroids; ADAMTS13 activity level was normal Following diagnosis of aHUS, eculizumab was initiated	Clinical signs improved in first week Creatinine normalized after 2 doses of maintenance eculizumab treatment Eculizumab was discontinued after 6 months and no signs of aHUS were observed 1 year after diagnosis
Mussoni et al. [38]	26-year-old female with strong family history of aHUS Diagnosed with aHUS and homozygous *CFH* mutation During first pregnancy, developed hypertension, hemolysis, proteinuria at approximately 12 weeks’ gestation; 1 month later, her clinical condition worsened (platelet count, 83 × 10^9^/L; LDH level, 380 U/L; hemoglobin level, 11.1 g/dL; proteinuria) Resolution of hemolytic parameters with PE but the patient could not discontinue without worsening of hemolysis, although renal function was normal Eculizumab was initiated	Normalization of hematologic abnormalities and reduction in proteinuria after 5 days of treatment Eculizumab was well tolerated without side effects Healthy newborn delivered via cesarean section at 38 weeks’ gestation Patient continued eculizumab therapy during and following pregnancy with no additional TMA
De Souza Amorim et al. [39]	41-year-old female admitted 4 days after childbirth for edema, asthenia, and severe hypertension Laboratory tests revealed thrombocytopenia, hemolytic anemia, and renal impairment; dialysis was initiated After differential diagnosis, aHUS was diagnosed and daily PE was initiated on day 7; the patient had hematologic normalization but no renal improvement Eculizumab was initiated on day 12, and PE was discontinued Patient determined to be homozygous carrier for *CFH* and *MCP* risk haplotypes	After 4 days on therapy, renal function improved and dialysis was discontinued Eculizumab was discontinued after 11 months and the patient has had good outcomes after 1 additional year of follow-up
Saad et al. [40]	19-year-old required labor induction at 39 weeks’ gestation, and was diagnosed with preeclampsia She had an uncomplicated delivery but developed signs of suspected HELLP syndrome on postpartum day 1 Laboratory findings (thrombocytopenia, hemolytic anemia, renal impairment) indicated TMA and the patient initiated PE After ADAMTS13 activity level was determined to be normal, aHUS was presumed and PE was discontinued The patient initiated eculizumab and an *MCP* mutation was later identified	Eculizumab was well tolerated and the patient had no additional signs of TMA
Tsai et al. [41]	20-year-old female with hypertension at 35 weeks’ gestation (second pregnancy) and history of gestational hypertension during first pregnancy 3 days after cesarean delivery, patient developed anasarca, confusion, seizures, and posterior reversible encephalopathy syndrome Laboratory tests revealed thrombocytopenia, hemolytic anemia, and renal impairment, which resolved over 5 weeks of daily PE; labetalol and nifedipine were required for hypertension control The patient’s third pregnancy at age 22 was also associated with hypertension, signs of TMA, and visual scotomas; her visual signs persisted following urgent delivery via induction aHUS with biopsy-proven TMA was diagnosed after rule out of TTP, and eculizumab was initiated Later, a *CFH* mutation was identified	With complement inhibition, the patient’s thrombocytopenia and symptoms resolved within 3 days Renal function normalized over 3 months
Hypertension/malignant hypertension		
Al-Akash et al. [56]	Male patient with history of aHUS and renal transplantation underwent second and third transplantations at 8 and 15 years of age due to TMA and allograft dysfunction Approximately 8 weeks post-transplant, the patient experienced an influenza infection, hypertension, fluid retention, and signs of TMA (thrombocytopenia and increasing LDH level) confirmed by renal biopsy Patient initiated PE and then initiated eculizumab therapy	On eculizumab, biopsies 6 and 13 months post-transplant showed improvement in TMA; clinical signs and symptoms also normalized BP was managed with only 1 antihypertensive
Garjau et al. [57]	44-year-old male with diarrhea, fever, and anuria; clinical and laboratory evaluation revealed BP of 220/150 mmHg, hemolytic anemia, abnormal LDH, and acute renal failure Patient began receiving PE/PI and dialysis Negative stool test for Shiga toxin and 57 % ADAMTS13 activity ruled out STEC-HUS and TTP, respectively; diagnosis of aHUS was confirmed with the discovery of an *MCP* mutation Renal biopsy confirmed TMA	After initiation of eculizumab, the patient had recovery of renal function and hematologic parameters; dialysis was discontinued BP was improved, although antihypertensives were still required Biopsy confirmed resolution of TMA
Besbas et al. [58]	3-day-old male infant with jaundice; developed macroscopic hematuria, severe hypertension (150/90 mmHg), thrombocytopenia, hemolytic anemia, increased LDH and serum creatinine levels, hematuria, and proteinuria *CFH* mutation confirmed diagnosis of aHUS PE/PI was initiated; hemodialysis was also required to stabilize renal function Patient experienced additional TMA manifestations at 1, 3 and 6 months of age, required increased use of PE/PI and dialysis; life-threatening hypertension required 5 antihypertensive agents	After initiation of eculizumab, patient had rapid recovery of hematologic parameters, renal function, and BP
Sajan et al. [59]	24-year-old male with 5-day history of nausea, vomiting, and mild diarrhea Physical examination revealed pulse rate of 95 beats per minute, BP of 156/96 mmHg, and appearance of mild dehydration; laboratory findings included thrombocytopenia, hemolytic anemia, and increased serum creatinine level; renal biopsy revealed evidence of TMA aHUS was diagnosed and the patient initiated PE; hemodialysis was required beginning on day 3 for worsening renal function and ongoing TMA Eculizumab was initiated and PE was discontinued on day 6; dialysis was discontinued at week 3 Hypertension was managed with a single antihypertensive; on day 58 the patient experienced accelerated hypertension and generalized tonic-clonic seizures; MRI revealed posterior reversible encephalopathy syndrome, which was managed with antiepileptics and antihypertensives	On eculizumab and 3 antihypertensives, the patient has had no further TMA manifestations, seizures, or hypertensive crises
Ohta et al. [60]	Severely ill 4-month-old male with fever and vomiting; laboratory testing revealed schistocytes, thrombocytopenia, elevated LDH, creatinine, and urea Diagnosis of aHUS made based on negative STEC test and 72 % ADAMTS13 activity Patient completed 2 weeks of PE/PI and dialysis with no improvement in hemolysis or renal failure Patient also developed severe hypertension (systolic BP of 140‒150 mmHg), which was refractory to nicardipine, enalapril, and losartan	After initiation of eculizumab, the patient’s hypertension and renal function improved and dialysis was discontinued Patient had 1 episode of cholestatic jaundice and was diagnosed with cholelithiasis, which resolved without treatment
Sevinc et al. [61]	32-year-old female with history of hypertension, proteinuria, and edema during a pregnancy 1 year prior and family history of TMA, presented with pyrexia, headache, tachycardia, and hypertension (160/110 mmHg) Fundoscopy revealed grade IV hypertensive retinopathy; other clinical and laboratory findings included mild pretibial and periorbital edema, oliguria, thrombocytopenia, and hemolytic anemia STEC-HUS and TTP were ruled out and the patient was diagnosed with aHUS; genetic analysis eventually revealed a *CFH* mutation PE initially improved hematologic values, which then worsened after 22 sessions	After initiating eculizumab and discontinuing PE, her hematologic values improved Dialysis was discontinued after 2 months of therapy Eculizumab was well tolerated
Sharma et al. [62]	28-day-old female with gross hematuria; physical and laboratory examinations revealed BP of 127/65 mmHg, thrombocytopenia, hemolytic anemia, increased serum creatinine level, and proteinuria Patient initiated daily PI on day 2 and dialysis on day 3 due to worsening renal function Patient experienced acute respiratory failure and hypothermia; there was no evidence of infection but dialysis was discontinued; echocardiogram revealed moderately reduced biventricular function Patient was intubated and dialysis was resumed; despite PE, she required RBC and platelet transfusions ADAMTS13 level was 76 % and aHUS was diagnosed; it was eventually determined that the patient had a *CFH* mutation	After initiation of eculizumab therapy, the patient discontinued dialysis within 4 days and had hematologic improvements within 5 days At 12 months of age, the patient is receiving ongoing eculizumab and propranolol for supraventricular tachycardia with normal renal function and BP
Tsai et al. [52]	49-year-old male with gross hematuria, coughing, dyspnea, abdominal pain, and vomiting Patient had fluctuating blood pressure and lethargy; history was notable for severe and unstable hypertension Laboratory tests revealed thrombocytopenia, hemolytic anemia, and renal failure aHUS was presumed and eculizumab was initiated	Within 1 week of therapy initiation, platelet count, extrarenal symptoms, and mental status resolved BP stabilized after 2 weeks, and renal function improved slowly over 6 weeks
Systemic lupus erythematosus		
Coppo et al. [79]	4-year-old female with SLE and diffuse proliferative lupus nephritis Developed worsening of general condition, along with abnormal hematologic (platelet count, LDH, hemoglobin, and haptoglobin) and renal (proteinuria and decrease in eGFR) as well as cardiovascular, neurologic, and pulmonary signs and symptoms Negative for common gene mutations associated with aHUS Treated with rituximab (no response)	On eculizumab, the patient had rapid disappearance of pulmonary symptoms and vasculitis as well as hematologic normalization and renal recovery TMA manifestations occurred after eculizumab discontinuation; the patient recovered after reintroduction of eculizumab therapy
El-Husseini et al. [80]	24-year-old female with 5-year history of SLE and lupus nephritis Developed hemolytic anemia, thrombocytopenia, and acute kidney injury; biopsy showed evidence of TMA aHUS suspected due to lupus nephritis Patient was treated with cyclophosphamide (for lupus nephritis) and received plasmapheresis and high-dose methylprednisolone with no response	Normalization of hematologic laboratory values and renal recovery on eculizumab therapy over a 6-month period, followed by therapy discontinuation
Hadaya et al. [83]	27-year-old female with ESRD Patient had biopsy evidence of severe TMA, complete glomerular scarring, and diffuse tubulointerstitial fibrosis Diagnosed with SLE with antiplatelet antibodies, lupus nephritis with fulminant TMA Negative for common gene mutations associated with aHUS Patient underwent renal transplantation after 10 months of dialysis TMA persisted after transplantation Patient received PE and 1 dialysis session, with no response	Improvement in symptoms and renal function with eculizumab Biopsy demonstrated inhibition of TMA on therapy
Ulcerative colitis		
Green et al. [96]	27-year-old female diagnosed 4 years prior with UC and primary sclerosing cholangitis; treated with 6-mercaptopurine and prednisone for multiple flares Presented with microangiopathic hemolytic anemia, acute kidney injury, watery diarrhea, and hypertension Abnormal laboratory findings included thrombocytopenia, high LDH and serum creatinine levels, and proteinuria Patient received 12 sessions of PE/PI with improvements in hematologic parameters but not renal function Eculizumab initiated; signs of TMA and serum creatinine normalized; evidence of complement activity (CH50, 96 %) briefly occurred during CMV colitis aHUS diagnosis further confirmed by identification of CFH autoantibodies	Patient is receiving ongoing eculizumab therapy with low-dose corticosteroids for inflammatory bowel disease No additional signs of TMA
Webb et al. [97]	16-year-old male with 4-year history of chronic active UC; flare 3 months prior and flu-like illness with high fever 2 months prior to presentation Presented with severe anemia, thrombocytopenia, hemolysis, and acute kidney injury Received packed RBC transfusion, methylprednisolone; discontinued 6-mercaptopurine (previously prescribed for flare) Clinical symptoms improved but thrombocytopenia and LDH and hemoglobin levels worsened; serum creatinine level remained elevated with evidence of proteinuria Biopsy findings were consistent with TMA; aHUS was diagnosed and eculizumab was initiated	On eculizumab, hematologic and renal parameters resolved Patient experienced brief UC flare that resolved with no additional therapy Patient resumed full activity including school attendance and sports activities

*ADAMTS13* a disintegrin and metalloproteinase with a thrombospondin type 1 motif, member 13, *aHUS* atypical hemolytic uremic syndrome, *BP* blood pressure, *CAC* complement-amplifying condition, *CAE* complement activity enzyme, *CFH* complement factor H, *CFI* complement factor I, *CMV* cytomegalovirus, *eGFR* estimated glomerular filtration rate, *ESRD* end-stage renal disease, *IV* intravenous, *LDH* lactate dehydrogenase, *MCP* membrane co-factor protein, *MRI* magnetic resonance imaging, *PE/PI* plasma exchange/plasma infusion, *RBC* red blood cell, *SLE* systemic lupus erythematosus, *STEC* Shiga-toxin producing *Escherichia coli, TMA* thrombotic microangiopathy, *TTP* thrombotic thrombocytopenic purpura, *UC* ulcerative colitis

Emerging evidence shows the safety of eculizumab during pregnancy despite potential placental transfer to the fetus. In a study of 75 pregnancies in 61 women with paroxysmal nocturnal hemoglobinuria (PNH) treated with eculizumab during pregnancy and postpartum, fetal mortality rates were not increased [38]. In these patients, eculizumab was present at low levels in 35 % of cord blood samples, but not in breast milk [42]. Similarly, recently reported case series involving pregnant PNH patients treated with eculizumab demonstrated low levels of the drug in cord blood, but not in breast milk [43, 44]. There were no adverse effects on the newborns noted.

#### Malignant hypertension

MHT can be associated with TMA [45, 46]. Many patients with aHUS first present with hypertension, potentially with high severity and/or MHT [7, 9, 10]. In a retrospective study of 45 children with aHUS, 71 % presented with hypertension [10]. In a large observational study, 8 % of patients with aHUS also had MHT [9].

The role of the endothelium as a pathogenic link between MHT and aHUS was recently reviewed [47]. TMA may occur following fluid shear stress on endothelial cells and subsequent vascular injury (i.e. fibrinoid necrosis, thrombosis, and luminal narrowing), leading to red blood cell fragmentation and platelet consumption [45, 46, 48]. Aldosterone has been implicated as a potential mediator of vascular endothelial damage in hypertension [21, 49, 50]. In one study, serum aldosterone levels were found to be higher in MHT patients with TMA versus those without TMA [21]. In addition, hypertensive crises are known to be prothrombotic, leading to platelet aggregation, thrombin generation, and fibrinolysis [51].

Patients with MHT may present with microangiopathic hemolytic anemia, renal impairment, and thrombocytopenia [46], although the latter may be modest and/or resolve quickly [26, 52]. Differentiation between MHT-associated TMA and TTP may be particularly difficult because both are associated with neurologic symptoms; however, renal dysfunction may be more common in TMA caused by MHT [26]. Prior history of hypertension and/or relatively high arterial pressure, signs of hypertensive heart disease, relatively high platelet count, and retinopathy are suggestive of MHT-associated TMA [26]. To that end, imaging techniques may be useful in confirming congestive heart disease and/or neurologic involvement due to MHT.

Without proper diagnosis and adequate treatment, patients with aHUS and hypertension and/or MHT may have severe symptoms and poor outcomes, including death [53, 54]. Because standard therapies for MHT do not address underlying complement dysregulation, TMA may persist despite such treatment. In a retrospective analysis of 21 patients with TMA and severe/malignant hypertension, 86 % did not recover normal renal function despite antihypertensive therapy [55]. It has been proposed that a diagnosis of aHUS should be suspected in patients with difficult-to-control MHT who demonstrate persistent TMA [52]. In such patients, treatment with eculizumab should be considered. Indeed, case studies have demonstrated the efficacy and safety of eculizumab, with or without antihypertensive agents, in the treatment aHUS in patients with MHT (Table 1) [52, 56–62].

#### Renal transplantation

Recurrent and de novo aHUS following renal transplantation have been reviewed in detail previously [5, 63]. The availability of eculizumab has substantially changed the landscape of renal transplantation in patients with aHUS [5, 63]. In the pre-eculizumab era, renal transplantation in aHUS was associated with poor graft survival and high rates of disease recurrence [64]. In contrast, a large series of 22 patients demonstrated that eculizumab therapy was effective in preventing and treating aHUS recurrence post-transplant [65]. Patients at high risk for recurrence are now candidates for renal transplantation [5]. Additionally, living-non-related donor transplantation may now be considered for certain patients [63]. Expert groups recommend that patients, especially with moderate or high risk for disease recurrence following renal transplantation, receive prophylactic eculizumab therapy [5, 63]. Eculizumab should also be considered for patients with de novo aHUS following renal transplantation [5].

#### Autoimmune diseases

##### Systemic lupus erythematosus

SLE is characterized by the formation of immune complexes that activate complement, leading to cellular injury [66]. Dysregulation of the terminal complement activation has been implicated in the pathogenesis and prognosis of SLE and lupus nephritis [66–68]. Complement gene mutations have been identified in patients with SLE and are associated with disease susceptibility [69] and earlier disease onset [69, 70]. In patients with SLE, TMA is associated with increased SLE activity, intercurrent infections [71], reduced long-term renal function, and poor overall survival [72–76]. Although TMA is typically Coombs-negative [5], patients with SLE and aHUS may have positive Coombs tests [77].

Recent case studies of SLE comorbid with aHUS have reported varying outcomes with standard SLE therapies (e.g. cyclophosphamide, high-dose steroids, mycophenolate mofetil). Patients may have slow recovery [78], no response [79–81], or remain dialysis-dependent [82]. Findings from reports of patients with SLE and aHUS treated with eculizumab have demonstrated the terminal complement inhibitor to be well tolerated and associated with improvement in symptoms, hematologic laboratory values, and renal function (Table 1) [79, 80, 83].

##### Scleroderma

Progressive scleroderma, or systemic sclerosis, can be complicated by chronic kidney disease associated with hypertension and mild proteinuria as well as by scleroderma renal crisis (SRC). SRC is the most severe form of renal disease in progressive scleroderma and carries a high mortality. SRC manifests as MHT, TMA, and rapid renal failure [84]. Diagnosis can be complicated by the lack of skin changes in some cases making renal histology and serology results the primary basis for appropriate diagnosis [85]. The pathogenesis of progressive scleroderma and its relation to aHUS is not well understood; it is believed that systemic vasoconstriction leads to ischemic injury and organ dysfunction [86].

Several cases of scleroderma-related aHUS have been reported in the literature [86–90]. Overall, outcomes were poor, including death within months of onset in one case [89]. The effects of eculizumab have not been documented in scleroderma-related aHUS. However, in a recent case report of SRC, in which diagnosis of aHUS was not ruled out or substantiated, treatment with eculizumab was associated with improvement in renal function, hypertension, and other symptoms [91].

##### Ulcerative colitis

Diagnosis of aHUS in patients with gastrointestinal symptoms may require differentiation from inflammatory bowel disorders such as ulcerative colitis (UC) [92]. Interestingly, alternative complement activation may also contribute to the pathogenesis of inflammatory bowel disorders. For example, it has been postulated that the upregulation of complement components may contribute to local inflammation and tissue damage in Crohn’s disease [93]. Inflammatory bowel disorders have been associated with upregulation of C3, which was strongly correlated with mucosal inflammation [94]. Deposition of C3b and the terminal complement complex have also been demonstrated in mucosal tissue from patients with UC [95].

Only 2 cases of UC-associated aHUS have been reported in the literature (Table 1) [96, 97]. In the case reported by Green et al. [96], the patient was found to have complement factor H autoantibodies. Both patients were treated with eculizumab and had favorable outcomes with improvement in renal function and hematologic parameters [96, 97].

#### Drug-induced TMA

aHUS and other TMAs may develop subsequent to the use of certain medications and have been reviewed elsewhere [5]. Data from a recent systematic review showed that nine medications account for 76 % of TMA cases: quinine, tacrolimus, cyclosporine, interferons, gemcitabine, mitomycin, clopidogrel, estrogen/progesterone, and ticlopidine [98]. The pathogenesis of drug-induced TMA involves two distinct mechanisms: immune mediated and direct toxicity. Evidence shows that quinine-, ticlopidine-, and clopidogrel-induced TMAs occur via an immune-mediated reaction, which is typically characterized by severe systemic manifestations including anuric acute kidney injury. TMA caused by cyclosporine, gemcitabine, and mitomycin occurs through a toxicity-mediated mechanism that is dose dependent and may also lead to renal impairment [98, 99]. For TMA caused by cancer medications, onset may or may not be dose related and timing can vary from immediately following therapy initiation to a 12-month delay [100]. Gemcitabine- and mitomycin-related TMA occur in <1 and 2‒15 % of patients, respectively, and outcomes may be quite poor: renal failure and/or mortality have been reported in up to 70–75 % of cases [101].

It is recommended that patients discontinue the medication if it is suspected to be the cause of TMA [1]. This approach should result in TMA resolution. Patients with TMA caused by immunosuppressants (e.g. cyclosporine, tacrolimus) should receive reduced doses or switch to another agent [102]. However, TMA may continue to progress despite the removal of an offending agent, as has been documented with both mitomycin- [103] and tacrolimus-induced TMA [104]. The optimal management strategy and timing for drug-induced TMA has not been established, but it has been suggested that drug avoidance and supportive care may be the only beneficial options [1]. However, the role for PE/PI is unclear [100], and outcomes have included lack of improvement in or worsening renal function in mitomycin- [104], tacrolimus- [104, 105], and gemcitabine-induced TMA [106]. In these example cases, eculizumab therapy, sometimes in addition to anti-inflammatory agents, led to improvements in both hematologic parameters and renal function [103–106]. More studies are necessary to determine a potential role for eculizumab in drug-induced TMA.

#### Infection-induced TMA

Infections, particularly of the respiratory and gastrointestinal tract, precede aHUS in approximately half of cases [4, 9]. Common bacterial and viral infections associated with TMA have been reviewed elsewhere [5, 63] and include *Streptococcus pneumoniae*, cytomegalovirus, H1N1 influenza, human immunodeficiency virus, and parvovirus. Such infections are thought to activate the alternative pathway of complement and may be associated with increased production of C5 and deposition of C5b-9 [63]. Clinical symptoms of some infections, including diarrhea, may complicate the diagnostic process [3, 5].

### Diagnosis and management of TMA in patients with CACs

aHUS can be clinically identical to other TMAs, including STEC-HUS and TTP. No testing is available for definitive diagnosis of aHUS [5]. In the setting of CACs and TMA, it may be particularly challenging to rule out other potential causes of TMA, including the existing CAC, to arrive at a diagnosis of aHUS [63]. It is possible that at the onset of aHUS, some patients with existing CACs may not present with all of the classic signs (i.e. thrombocytopenia, microangiopathic hemolytic anemia, acute renal failure) [53].

An algorithm for the management of patients with CACs and TMA is presented in Fig. 1. In a patient presenting with TMA and a specific CAC [9, 20, 24, 28, 53], clinicians should first initiate specific management for the identified CAC, in order to treat underlying causes of TMA. If renal or extrarenal TMA arose from the CAC in the absence of complement dysregulation, TMA should resolve rapidly. As the French Study Group for aHUS/C3G (C3 glomerulopathies) has recommended, resolution of renal TMA can be defined as normalization of platelet count and LDH level, and a decrease in serum creatinine by ≥25 % [63].

The persistence of TMA despite specific management for the CAC strongly suggests that the CAC is lowering the threshold for manifestations and unmasking aHUS [5, 24, 35, 53]. In these cases, differential diagnosis of TMA is required [5]. All patients with TMA should have a thorough evaluation for underlying causes [5]. STEC-HUS can be ruled out with a negative stool test for STEC. ADAMTS13 activity <5 % (depending on the assay used) indicates TTP [5]. Genetic investigations may help determine long-term prognosis of aHUS but are not required for diagnosis [5]. Complement gene mutations or factor H autoantibodies are identified in approximately 50–70 % of patients with aHUS [4, 9]. The number of mutations characterized has been increasing steadily over recent years [107], and others may be identified in the future.

Overall, clinicians should have a strong suspicion for aHUS in patients with ADAMTS13 activity ≥5 %, negative STEC test, and persistent TMA despite treatment of the CAC. It should be noted that testing results for ADAMTS13 activity level and STEC may not be rapidly available. Thus, some patients may initiate PE during the differential diagnosis period [5] to temporarily maintain hematologic parameters, although it does not inhibit the underlying complement-mediated pathogenesis of aHUS [12] or prevent end-stage renal disease or mortality [9].

For patients with diagnosed aHUS, with or without a comorbid CAC, clinicians should initiate eculizumab therapy immediately according to established guidelines [5, 63, 108]. Clinical studies have shown that earlier intervention with eculizumab is associated with better renal outcomes for patients with aHUS [15]. The specific effects of eculizumab in aHUS comorbid with individual CACs (e.g. autoimmune diseases) will be demonstrated as evidence accumulates in the literature.

The optimal treatment duration for patients with aHUS and specific CACs has not been established. Expert and regulatory guidance notes that ongoing treatment may prevent risks of potentially life-threatening TMA that may occur following therapy discontinuation [5, 13, 14].

## Conclusions

CACs are increasingly identified in the medical literature as being comorbid with aHUS or unmasking previously undiagnosed cases. The presented case studies illustrate potential complexities in disease onset and differential diagnosis when both a CAC and aHUS are present, as well as the benefits of eculizumab treatment for these patients. Overall, clinicians should consider a diagnosis of aHUS if TMA persists despite specific management for the CAC. Once aHUS is diagnosed, eculizumab should be initiated promptly to halt target organ injury and improve outcomes related to TMA.

## References

[1] George JN, Nester CM (2014). Syndromes of thrombotic microangiopathy. N Engl J Med.

[2] Moake JL (2002). Thrombotic microangiopathies. N Engl J Med.

[3] Noris M, Remuzzi G (2009). Atypical hemolytic-uremic syndrome. N Engl J Med.

[4] Fremeaux-Bacchi V, Fakhouri F, Garnier A (2013). Genetics and outcome of atypical hemolytic uremic syndrome: a nationwide French series comparing children and adults. Clin J Am Soc Nephrol.

[5] Campistol JM, Arias M, Ariceta G (2015). An update for atypical haemolytic uraemic syndrome: diagnosis and treatment. A consensus document. Nefrologia.

[6] Nayer A, Asif A (2016). Atypical hemolytic-uremic syndrome: a clinical review. Am J Ther.

[7] Neuhaus TJ, Calonder S, Leumann EP (1997). Heterogeneity of atypical haemolytic uraemic syndromes. Arch Dis Child.

[8] Sellier-Leclerc AL, Fremeaux-Bacchi V, Dragon-Durey MA (2007). Differential impact of complement mutations on clinical characteristics in atypical hemolytic uremic syndrome. J Am Soc Nephrol.

[9] Noris M, Caprioli J, Bresin E (2010). Relative role of genetic complement abnormalities in sporadic and familial aHUS and their impact on clinical phenotype. Clin J Am Soc Nephrol.

[10] Geerdink LM, Westra D, van Wijk JA (2012). Atypical hemolytic uremic syndrome in children: complement mutations and clinical characteristics. Pediatr Nephrol.

[11] Ariceta G, Besbas N, Johnson S (2009). Guideline for the investigation and initial therapy of diarrhea-negative hemolytic uremic syndrome. Pediatr Nephrol.

[12] Loirat C, Garnier A, Sellier-Leclerc AL, Kwon T (2010). Plasmatherapy in atypical hemolytic uremic syndrome. Semin Thromb Hemost.

[13] US Food and Drug Administration (2015). Soliris (eculizumab) [prescribing information].

[14] European Medicines Agency (2015). Soliris (eculizumab) [summary of product characteristics].

[15] Legendre CM, Licht C, Muus P (2013). Terminal complement inhibitor eculizumab in atypical hemolytic-uremic syndrome. N Engl J Med.

[16] Licht C, Greenbaum LA, Muus P (2015). Efficacy and safety of eculizumab in atypical hemolytic uremic syndrome from 2-year extensions of phase 2 studies. Kidney Int.

[17] Greenbaum LA, Fila M, Ardissino G (2016). Eculizumab is a safe and effective treatment in pediatric patients with atypical hemolytic uremic syndrome. Kidney Int.

[18] Fakhouri F, Hourmant M, Campistol JM (2016). Terminal complement inhibitor eculizumab in adult patients with atypical hemolytic uremic syndrome: a single-arm, open-label trial. Am J Kidney Dis.

[19] Riedl M, Fakhouri F, Le Quintrec M (2014). Spectrum of complement-mediated thrombotic microangiopathies: pathogenetic insights identifying novel treatment approaches. Semin Thromb Hemost.

[20] Kavanagh D, Goodship THJ, Richards A (2006). Atypical haemolytic uraemic syndrome. Br Med Bull.

[21] Akimoto T, Muto S, Ito C (2011). Clinical features of malignant hypertension with thrombotic microangiopathy. Clin Exp Hypertens.

[22] Barbour T, Johnson S, Cohney S, Hughes P (2012). Thrombotic microangiopathy and associated renal disorders. Nephrol Dial Transplant.

[23] Nester CM, Thomas CP (2012). Atypical hemolytic uremic syndrome: what is it, how is it diagnosed, and how is it treated?. Hematology Am Soc Hematol Educ Program.

[24] Noris M, Mescia F, Remuzzi G (2012). STEC-HUS, atypical HUS and TTP are all diseases of complement activation. Nat Rev Nephrol.

[25] Kavanagh D, Goodship TH, Richards A (2013). Atypical hemolytic uremic syndrome. Semin Nephrol.

[26] Khanal N, Dahal S, Upadhyay S, Bhatt VR, Bierman PJ (2015). Differentiating malignant hypertension-induced thrombotic microangiopathy from thrombotic thrombocytopenic purpura. Ther Adv Hematol.

[27] Dashe JS, Ramin SM, Cunningham FG (1998). The long-term consequences of thrombotic microangiopathy (thrombotic thrombocytopenic purpura and hemolytic uremic syndrome) in pregnancy. Obstet Gynecol.

[28] Fakhouri F, Roumenina L, Provot F (2010). Pregnancy-associated hemolytic uremic syndrome revisited in the era of complement gene mutations. J Am Soc Nephrol.

[29] Vaught AJ, Gavriilaki E, Hueppchen N (2016). Direct evidence of complement activation in HELLP syndrome: a link to atypical hemolytic uremic syndrome. Exp Hematol.

[30] Shrivastava M, Modi G, Singh RK, Navaid S (2011). Early diagnosis and management of postpartum hemolytic uremic syndrome with plasma exchange. Transfus Apher Sci.

[31] Mu J, Zhang J, Sunnassee A, Dong H (2015). A case report of undiagnosed postpartum hemolytic uremic syndrome. Diagn Pathol.

[32] Calvert GD (1972). Postpartum haemolytic uraemic syndrome: case report and brief review. J Obstet Gynaecol Br Commonw.

[33] Delmas Y, Bordes C, Loirat C, Fremeaux-Bacchi V, Combe C (2013). Post-partum atypical haemolytic-uraemic syndrome treated with eculizumab: terminal complement activity assessment in clinical practice. Clin Kidney J.

[34] Ardissino G, Wally Ossola M, Baffero GM, Rigotti A, Cugno M (2013). Eculizumab for atypical hemolytic uremic syndrome in pregnancy. Obstet Gynecol.

[35] Carr R, Cataland SR (2013). Relapse of aHUS after discontinuation of therapy with eculizumab in a patient with aHUS and factor H mutation. Ann Hematol.

[36] Zschiedrich S, Prager EP, Kuehn EW (2013). Successful treatment of the postpartum atypical hemolytic uremic syndrome with eculizumab. Ann Intern Med.

[37] Canigral C, Moscardo F, Castro C (2014). Eculizumab for the treatment of pregnancy-related atypical hemolytic uremic syndrome. Ann Hematol.

[38] Mussoni MP, Veneziano FA, Boetti L (2014). Innovative therapeutic approach: sequential treatment with plasma exchange and eculizumab in a pregnant woman affected by atypical hemolytic-uremic syndrome. Transfus Apher Sci.

[39] De Sousa Amorim E, Blasco M, Quintana L, Sole M, de Cordoba SR, Campistol JM (2015). Eculizumab in pregnancy-associated atypical hemolytic uremic syndrome: insights for optimizing management. J Nephrol.

[40] Saad AF, Roman J, Wyble A, Pacheco LD (2016). Pregnancy-associated atypical hemolytic-uremic syndrome. AJP Rep.

[41] Tsai HM, Kuo E (2016). From gestational hypertension and preeclampsia to atypical hemolytic uremic syndrome. Obstet Gynecol.

[42] Kelly RJ, Hochsmann B, Szer J (2015). Eculizumab in pregnant patients with paroxysmal nocturnal hemoglobinuria. N Engl J Med.

[43] Hallstensen RF, Bergseth G, Foss S (2015). Eculizumab treatment during pregnancy does not affect the complement system activity of the newborn. Immunobiology.

[44] Miyasaka N, Miura O, Kawaguchi T (2016). Pregnancy outcomes of patients with paroxysmal nocturnal hemoglobinuria treated with eculizumab: a Japanese experience and updated review. Int J Hematol.

[45] Shibagaki Y, Fujita T (2005). Thrombotic microangiopathy in malignant hypertension and hemolytic uremic syndrome (HUS)/thrombotic thrombocytopenic purpura (TTP): can we differentiate one from the other?. Hypertens Res.

[46] van den Born BJ, Honnebier UP, Koopmans RP, van Montfrans GA (2005). Microangiopathic hemolysis and renal failure in malignant hypertension. Hypertension.

[47] Mathew RO, Nayer A, Asif A (2016). The endothelium as the common denominator in malignant hypertension and thrombotic microangiopathy. J Am Soc Hypertens.

[48] Kincaid-Smith P (1982). Renal pathology in hypertension and the effects of treatment. Br J Clin Pharmacol.

[49] Farquharson CA, Struthers AD (2002). Aldosterone induces acute endothelial dysfunction in vivo in humans: evidence for an aldosterone-induced vasculopathy. Clin Sci (Lond).

[50] Cachofeiro V, Miana M, de Las Heras N (2008). Aldosterone and the vascular system. J Steroid Biochem Mol Biol.

[51] van den Born BJH, Lowenberg EC, van der Hoeven NV (2011). Endothelial dysfunction, platelet activation, thrombogenesis and fibrinolysis in patients with hypertensive crisis. J Hypertens.

[52] Tsai HM (2016). Does anticomplement therapy have a role in the management of malignant hypertension?. J Clin Hypertens (Greenwich).

[53] Totina A, Iorember F, El-Dahr SS, Yosypiv IV (2013). Atypical hemolytic-uremic syndrome in a child presenting with malignant hypertension. Clin Pediatr (Phila).

[54] Rafiq A, Tariq H, Abbas N, Shenoy R (2015). Atypical hemolytic-uremic syndrome: a case report and literature review. Am J Case Rep.

[55] Zhang B, Xing C, Yu X, Sun B, Zhao X, Qian J (2008). Renal thrombotic microangiopathies induced by severe hypertension. Hypertens Res.

[56] Al-Akash SI, Almond PS, Savell VH, Gharaybeh SI, Hogue C (2011). Eculizumab induces long-term remission in recurrent post-transplant HUS associated with C3 gene mutation. Pediatr Nephrol.

[57] Garjau M, Azancot M, Ramos R, Sanchez-Corral P, Montero MA, Seron D (2012). Early treatment with eculizumab in atypical haemolytic uraemic syndrome. Clin Kidney J.

[58] Besbas N, Gulhan B, Karpman D (2013). Neonatal onset atypical hemolytic uremic syndrome successfully treated with eculizumab. Pediatr Nephrol.

[59] Sajan T, Vinay S, Sonu N, Alan P (2014). How atypical can atypical hemolytic uremic syndrome be?. Clin Case Rep.

[60] Ohta T, Urayama K, Tada Y (2015). Eculizumab in the treatment of atypical hemolytic uremic syndrome in an infant leads to cessation of peritoneal dialysis and improvement of severe hypertension. Pediatr Nephrol.

[61] Sevinc M, Basturk T, Sahutoglu T (2015). Plasma resistant atypical hemolytic uremic syndrome associated with a *CFH* mutation treated with eculizumab: a case report. J Med Case Rep.

[62] Sharma S, Pradhan M, Meyers KE, Le Palma K, Laskin BL (2015). Neonatal atypical hemolytic uremic syndrome from a factor H mutation treated with eculizumab. Clin Nephrol.

[63] Zuber J, Fakhouri F, Roumenina LT, Loirat C, Fremeaux-Bacchi V (2012). Use of eculizumab for atypical haemolytic uraemic syndrome and C3 glomerulopathies. Nat Rev Nephrol.

[64] Le Quintrec M, Zuber J, Moulin B (2013). Complement genes strongly predict recurrence and graft outcome in adult renal transplant recipients with atypical hemolytic and uremic syndrome. Am J Transplant.

[65] Zuber J, Le Quintrec M, Krid S (2012). Eculizumab for atypical hemolytic uremic syndrome recurrence in renal transplantation. Am J Transplant.

[66] Ornstein BW, Atkinson JP, Densen P (2012). The complement system in pediatric systemic lupus erythematosus, atypical hemolytic uremic syndrome, and complocentric membranoglomerulopathies. Curr Opin Rheumatol.

[67] Leffler J, Bengtsson AA, Blom AM (2014). The complement system in systemic lupus erythematosus: an update. Ann Rheum Dis.

[68] Birmingham DJ, Hebert LA (2015). The complement system in lupus nephritis. Semin Nephrol.

[69] Zhao J, Wu H, Khosravi M (2011). Association of genetic variants in complement factor H and factor H-related genes with systemic lupus erythematosus susceptibility. PLoS Genet.

[70] Jonsen A, Nilsson SC, Ahlqvist E (2011). Mutations in genes encoding complement inhibitors *CD46* and *CFH* affect the age at nephritis onset in patients with systemic lupus erythematosus. Arthritis Res Ther.

[71] Chen MH, Chen MH, Chen WS (2011). Thrombotic microangiopathy in systemic lupus erythematosus: a cohort study in North Taiwan. Rheumatology (Oxford).

[72] Bridoux F, Vrtovsnik F, Noel C (1998). Renal thrombotic microangiopathy in systemic lupus erythematosus: clinical correlations and long-term renal survival. Nephrol Dial Transplant.

[73] Song D, Wu LH, Wang FM (2013). The spectrum of renal thrombotic microangiopathy in lupus nephritis. Arthritis Res Ther.

[74] Musio F, Bohen EM, Yuan CM, Welch PG (1998). Review of thrombotic thrombocytopenic purpura in the setting of systemic lupus erythematosus. Semin Arthritis Rheum.

[75] Banfi G, Bertani T, Boeri V (1991). Renal vascular lesions as a marker of poor prognosis in patients with lupus nephritis. Gruppo Italiano per lo Studio della Nefrite Lupica (GISNEL). Am J Kidney Dis.

[76] Jain R, Chartash E, Susin M, Furie R (1994). Systemic lupus erythematosus complicated by thrombotic microangiopathy. Semin Arthritis Rheum.

[77] Giannouli S, Voulgarelis M, Ziakas PD, Tzioufas AG (2006). Anaemia in systemic lupus erythematosus: from pathophysiology to clinical assessment. Ann Rheum Dis.

[78] Samson M, Audia S, Leguy V (2012). Haemolytic-uraemic syndrome during severe lupus nephritis: efficacy of plasma exchange. Intern Med J.

[79] Coppo R, Peruzzi L, Amore A (2015). Dramatic effects of eculizumab in a child with diffuse proliferative lupus nephritis resistant to conventional therapy. Pediatr Nephrol.

[80] El-Husseini A, Hannan S, Awad A, Jennings S, Cornea V, Sawaya BP (2015). Thrombotic microangiopathy in systemic lupus erythematosus: efficacy of eculizumab. Am J Kidney Dis.

[81] Ramachandran R, Sakhuja V, Jha V, Kohli HS, Rathi M (2012). Plasmapheresis in systemic lupus erythematosus with thrombotic microangiopathy. Intern Med J.

[82] Gharbi C, Bourry E, Rouvier P (2010). Rapidly progressive lupus nephritis and concomitant thrombotic microangiopathy. Clin Exp Nephrol.

[83] Hadaya K, Ferrari-Lacraz S, Fumeaux D (2011). Eculizumab in acute recurrence of thrombotic microangiopathy after renal transplantation. Am J Transplant.

[84] Stratta P, Besso L, Ferrero S (1996). Scleroderma renal crisis is still a life-threatening syndrome. Ren Fail.

[85] Zwettler U, Andrassy K, Waldherr R, Ritz E (1993). Scleroderma renal crisis as a presenting feature in the absence of skin involvement. Am J Kidney Dis.

[86] Yamanaka K, Mizutani H, Hashimoto K, Nishii M, Shimizu M (1997). Scleroderma renal crisis complicated by hemolytic uremic syndrome in a case of elderly onset systemic sclerosis. J Dermatol.

[87] Ricker DM, Sharma HM, Nahman NS (1989). Acute renal failure with glomerular thrombosis in a patient with chronic scleroderma. Am J Kidney Dis.

[88] Meyrier A, Becquemont L, Weill B, Callard P, Rainfray M (1991). Hemolytic-uremic syndrome with anticardiolipin antibodies revealing paraneoplastic systemic scleroderma. Nephron.

[89] Haviv YS, Safadi R (1998). Normotensive scleroderma renal crisis: case report and review of the literature. Ren Fail.

[90] Chen WS, Young AH, Wang HP, Huang DF (2009). Hemolytic uremic syndrome with ischemic glomerulonephropathy and obliterative vasculopathy in a systemic sclerosis patient treated with cyclosporine-A. Rheumatol Int.

[91] Thomas CP, Nester CM, Phan AC, Sharma M, Steele AL, Lenert PS (2015). Eculizumab for rescue of thrombotic microangiopathy in PM-Scl antibody-positive autoimmune overlap syndrome. Clin Kidney J.

[92] Craner GE, Burdick GE (1976). Acute colitis resembling ulcerative colitis in the hemolytic-uremic syndrome. Am J Dig Dis.

[93] Ahrenstedt O, Knutson L, Nilsson B, Nilsson-Ekdahl K, Odlind B, Hallgren R (1990). Enhanced local production of complement components in the small intestines of patients with Crohn’s disease. N Engl J Med.

[94] Sugihara T, Kobori A, Imaeda H (2010). The increased mucosal mRNA expressions of complement C3 and interleukin-17 in inflammatory bowel disease. Clin Exp Immunol.

[95] Halstensen TS, Mollnes TE, Garred P, Fausa O, Brandtzaeg P (1992). Surface epithelium related activation of complement differs in Crohn’s disease and ulcerative colitis. Gut.

[96] Green H, Harari E, Davidovits M (2014). Atypical HUS due to factor H antibodies in an adult patient successfully treated with eculizumab. Ren Fail.

[97] Webb TN, Griffiths H, Miyashita Y (2015). Atypical hemolytic uremic syndrome and chronic ulcerative colitis treated with eculizumab. Int J Med Pharm Case Reports.

[98] Al-Nouri ZL, Reese JA, Terrell DR, Vesely SK, George JN (2015). Drug-induced thrombotic microangiopathy: a systematic review of published reports. Blood.

[99] Reese JA, Bougie DW, Curtis BR (2015). Drug-induced thrombotic microangiopathy: experience of the Oklahoma registry and the BloodCenter of Wisconsin. Am J Hematol.

[100] Izzedine H, Perazella MA (2015). Thrombotic microangiopathy, cancer, and cancer drugs. Am J Kidney Dis.

[101] Blake-Haskins JA, Lechleider RJ, Kreitman RJ (2011). Thrombotic microangiopathy with targeted cancer agents. Clin Cancer Res.

[102] Pisoni R, Ruggenenti P, Remuzzi G (2001). Drug-induced thrombotic microangiopathy: incidence, prevention and management. Drug Saf.

[103] Faguer S, Huart A, Fremeaux-Bacchi V, Ribes D, Chaveau D (2013). Eculizumab and drug-induced haemolytic-uraemic syndrome. Clin Kidney J.

[104] Safa K, Logan MS, Batal I, Gabardi S, Rennke HG, Abdi R (2015). Eculizumab for drug-induced de novo posttransplantation thrombotic microangiopathy: a case report. Clin Nephrol.

[105] Nurnberger J, Philipp T, Witzke O (2009). Eculizumab for atypical hemolytic-uremic syndrome. N Engl J Med.

[106] Starck M, Wendtner CM (2014). Use of eculizumab in refractory gemcitabine-induced thrombotic microangiopathy. Br J Haematol.

[107] Noris M, Remuzzi G (2013). Managing and preventing atypical hemolytic uremic syndrome recurrence after kidney transplantation. Curr Opin Nephrol Hypertens.

[108] Loirat C, Fakhouri F, Ariceta G (2016). An international consensus approach to the management of atypical hemolytic uremic syndrome in children. Pediatr Nephrol.

